# Pulmonary Delivery of Anti-microRNA Oligonucleotide and Glycyrrhizic Acid Using Ternary Peptide Micelles for the Treatment of Acute Lung Injury

**DOI:** 10.34133/bmr.0107

**Published:** 2024-11-08

**Authors:** Minji Kang, Chuanyu Zhuang, Jihun Oh, Minhyung Lee

**Affiliations:** Department of Bioengineering, Hanyang University, 222 Wangsimni-ro, Seoul, Republic of Korea.

## Abstract

Acute lung injury (ALI) is a devastating inflammatory disease. In lungs with inflammation, microRNA155 (miR155) induces inflammatory cytokines by inhibiting the expression of suppressor of cytokine signaling-1 (SOCS1). In addition, glycyrrhizic acid (GA) has been suggested as an anti-inflammatory drug for ALI, since it is an efficient inhibitor of nuclear factor-κB. In this study, a combined delivery system of anti-miR155 oligonucleotides (AMO155) and GA was developed with R3V6 for the treatment of ALI. R3V6s formed comicelles with cholesterol-conjugated AMO155 (AMO155c) by charge and hydrophobic interactions. GA, an amphiphilic drug, was integrated to AMO155c-R3V6 micelles, producing AMO155c-R3V6-GA ternary micelles. The size of AMO155c-R3V6-GA was smaller than that of AMO155c-R3V6, suggesting that GA integration reduced the size of the micelles effectively. In addition, AMO155c-R3V6-GA had higher delivery efficiency than AMO155c-R3V6 micelles. In the comparison of AMO155-R3V6-GA and AMO155c-R3V6-GA, cholesterol moiety of AMO155c increased the stability and delivery efficiency of the ternary micelles. For in vivo evaluation, nebulized AMO155c-R3V6-GA micelle solution were administrated into the lungs of the ALI animal models intratracheally. AMO155c-R3V6-GA micelles had improved AMO155c delivery efficiency, compared with the AMO155c-polyethylenimine complex and AMO155c-R3V6 micelles in the lungs. As a result, SOCS1 expression was increased, and proinflammatory cytokines were reduced in the AMO155c-R3V6-GA micelle groups, compared with the other groups. In conclusion, AMO155c-R3V6-GA ternary micelles may be a useful delivery system for combined therapy of AMO155 and GA for the treatment of ALI.

## Introduction

Acute lung injury (ALI) is a devastating inflammatory disease of the lung. ALI is characterized by edema along with respiratory failure, lung infiltration, and hypoxia [1,2]. ALI has various causes such as infection, ischemia–reperfusion, and sepsis. Recently, COVID-19 has become one of the main causes of ALI. In ALI, the barrier permeability between the alveoli and capillaries increases, resulting in a leakage of protein-rich fluids into the air space [3]. Neutrophil infiltration into the lungs and secretion of proinflammatory cytokines induce an eruption of immune reactions, inducing a so-called “cytokine storm” [4–6]. The typical treatment for ALI is mechanical ventilation. Mechanical ventilation can be administered to patients flexibly, depending on whether their conditions are mild or severe [7]. Anti-inflammatory drugs such as diuretics and corticosteroids are also under investigation as therapeutic agents for ALI [8]. In clinical trials, the drugs reduced the stay time in an intensive care unit or the time using a ventilator. However, side effects, such as drug resistance, toxicity, and osteoporosis, limited their application in ALI [9]. Thus, more effective treatments should be developed for ALI.

Gene therapy has been studied as a therapeutic option for ALI. Therapeutic genes such as *adiponectin*, *heme oxygenase-1*, and *angiopoietin-1* have been transferred into the lungs using polymers, liposomes, or ultrasound [10,11]. The gene delivery had therapeutic effects, reducing inflammatory reactions in the ALI animal models. In addition, small oligonucleotides including small interfering RNA (siRNA) have been evaluated as therapeutic reagents. Anti-tumor necrosis factor-α (TNF-α) siRNAs down-regulated inflammatory reactions such as monocyte infiltration and cytokine release [12]. siRNAs against sphingosine 1-phosphate (anti-S1P siRNAs) were reported to maintain the vascular barrier integrity, resulting in decrease of inflammatory reaction [13]. Combination therapy of genes with anti-inflammatory drug was also reported to have improved therapeutic effects in ALI animal models. For example, RAGE antagonist peptide with the adiponectin gene decreased inflammatory reactions effectively, compared with gene therapy alone [14].

Although animal experiments with genes showed positive therapeutic effects, gene delivery into the lungs was challenging. The surface of lung epithelial cells is covered with a mucus layer to protect the cells [10,15,16]. Although the mucus layer is essential for protecting the lungs from infection or toxification, it is a barrier to the pulmonary delivery of genes by inhalation. Positively charged substances are easily captured by mucins in the mucus layer and cleared rapidly without reaching the lung epithelial cells [17]. Therefore, the surface charge of the therapeutic agent is one of the important factors affecting delivery efficiency. According to a previous report, particles with a slightly negative surface charge had the highest mucus penetrating effect, increasing delivery efficiency [18]. Liposomes and polymers used in gene delivery have a positive charge for complexation with negatively charged genes. To form a stable complex, excess amounts of liposomes or polymers are added to DNA, producing complexes with a positive surface charge. Although the complexes with positive surface charges are stable, they are not effective in penetrating the mucus layer. Therefore, masking the positive surface charges of the carriers is required for efficient gene delivery [19].

In the treatment of ALI, polymeric carriers have been investigated for pulmonary gene delivery [10]. The carriers include polyethylenimine (PEI), polyamidoamine (PAMAM), dexamethasone-conjugated PEI, and R3V6 [10]. In particular, R3V6 was investigated as a carrier of antisense oligonucleotides into the lungs [20,21]. Due to a hydrophobic V6 stretch and a hydrophilic R3 stretch, R3V6s form stable micelles in aqueous solution. R3V6 micelles form stable complexes with anti-microRNA oligonucleotides (AMOs) due to charge interactions between arginine residues and oligonucleotides [22]. In animal experiments, R3V6 had higher pulmonary delivery efficiency than PEI with a molecular weight of 25 kDa (PEI25k) [20]. However, they produce R3V6/AMO complexes with a positive surface charge, which may hamper delivery efficiency due to rapid clearance. The positive-surface-charged particles may induce toxicity to the lung cells. Therefore, a reduction in the positive charges of the R3V6/AMO complexes may be required to increase the efficiency of gene delivery.

Recent studies have found that some microRNAs (miRNAs) are induced in the lung cells of ALI and increase inflammation reactions [23]. MiRNAs can bind to their target mRNAs and inhibit their expression. One of them is miRNA155, which is rapidly induced in ALI lungs [24]. The miRNA155 inhibits the expression of the suppressor of cytokine signaling-1 (SOCS1) gene, an inhibitor of nuclear factor-κB (NF-κB) [25]. As a result, proinflammatory cytokines such as TNF-α and interleukin-1β (IL-1β) are induced by miRNA155, increasing inflammatory reactions [26,27]. Thus, it has been suggested that inhibition of miRNA155 may be a useful strategy to treat ALI. The anti-miRNA155 oligonucleotide (AMO155) is used as a therapeutic gene to suppress its target the microRNA155 sequence [21,28,29].

**Fig. 1.**
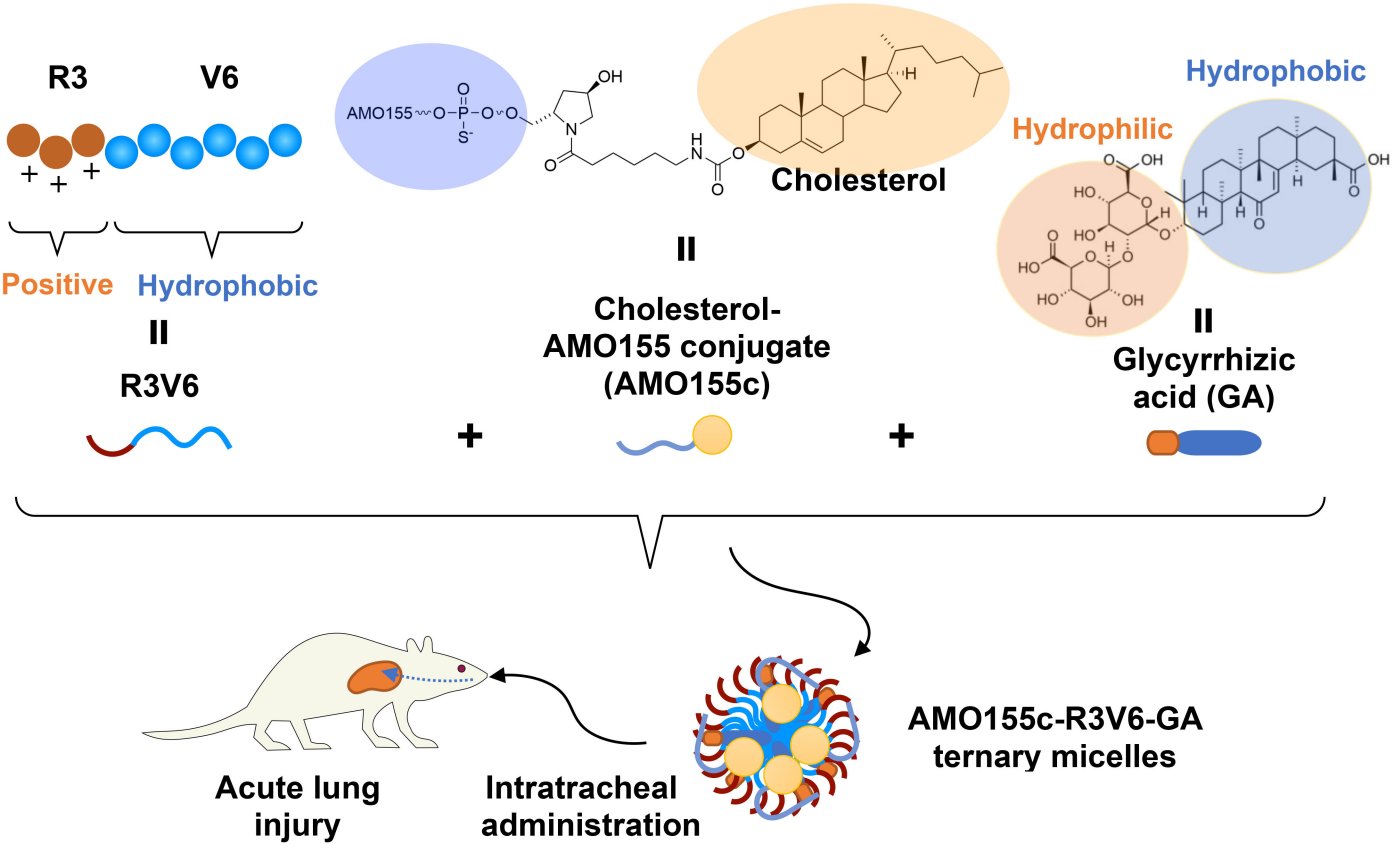
Schematic representation of AMO155c-R3V6-GA micelles.

In this study, we developed a novel codelivery system of AMO155 and anti-inflammatory drug, based on cholesterol-conjugated AMO155 (AMO155c). AMO155c has some advantages over AMO155 without cholesterol. First, AMO155c can form stable comicelles with R3V6 by hydrophobic interaction with the valines of R3V6 as well as charge interaction between AMO and arginines. Therefore, AMO155c may form stable nanoparticles with less amount of R3V6 than AMO155 without cholesterol. Second, amphiphilic anti-inflammatory drugs such as glycyrrhizic acid (GA) can be integrated into the comicelles, producing AMO155c-R3V6-GA ternary micelles (Fig. 1). GA is a natural substance from licorice root. It has antioxidant and anti-inflammatory effects through the inhibition of the NF-κB pathway [30,31]. In addition, GA is an amphiphilic compound with a negative charge, and therefore, integration of GA into the comicelles may decrease the positive surface charge of the comicelles, resulting in increase of mucus penetration efficiency. Therefore, AMO155c-R3V6-GA micelles may be an efficient carrier for codelivery of AMO155 and GA into the lungs. In this study, AMO155c-R3V6-GA micelles were characterized to determine micelle formation, surface charge, delivery efficiency into the cells, and anti-inflammatory effects. The therapeutic effects were also evaluated in lipopolysaccharide (LPS)-induced ALI animal models.

## Materials and Methods

### Materials

Raw264.7 and L2 cells were obtained from the Korean Cell Line Bank (Seoul, Korea). AMO155 (5′-ACCCCUAUCACAAUUAGCAUUAA-3′) and cholesterol-modified AMO155 (5′-ACCCCUAUCACAAUUAGCAUUAA-cholesterol-3′), scrAMO155c (5′-UCACAACCUCCUAGAAAGAGUA-cholesterol-3′), glyceraldehyde 3-phosphate dehydrogenase primers (forward: 5′-AGACAGCCGCATCTTCTTGT-3′, reverse: 5′-CTTGCCGTGGGTAGAGTCAT-3′), and miRNA155 primers (forward: 5′-ACTGTTAATGCTAATTGTGATAGG-3′, reverse: 5′-GTGCAGGGTCCGAGGTATTC-3′) were synthesized by Bioneer (Daejeon, Korea). Dulbecco’s modified Eagle medium, fetal bovine serum, and Roswell Park Memorial Institute 1640 (RPMI 1640) medium were obtained from Welgene (Seoul, Korea). R3V6 was chemically synthesized by Peptron (Daejeon, Korea). Enzyme-linked immunosorbent assay (ELISA) kits and Alexa Fluor 488-labeled goat anti-mouse immunoglobulin G secondary antibody was obtained from Invitrogen (Eugene, OR, USA). The kit for bicinchoninic acid assay was obtained from Thermo Scientific (Rockford, IL, USA). Amiloride hydrochloride hydrate (amiloride), 4′,6-diamidino-2-phenylindole (DAPI), chloroquine diphosphate salt (chloroquine), 1,6-diphenyl-1,3,5-hexatriene (DPH), heparin, GA, and methyl-β-cyclodextrin (MβCD) were obtained from Sigma-Aldrich (St. Louis, MO, USA). Chlorpromazine hydrochloride (chlorpromazine) and chloroquine diphosphate (chloroquine) were purchased from Tokyo Chemical Industry (Tokyo, Japan). SOCS1 antibody was obtained from Santa Cruz Biotechnology (Dallas, TX, USA).

### Preparation of AMO155c-R3V6-GA micelles

R3V6 and GA were combined at a designated weight ratio and dissolved in 5% glucose solution to a final concentration of 1 mg/ml. The resulting solution was subjected to water sonication at a power of 70 W for 20 min to ensure proper dispersion. Subsequently, AMO155c was added dropwise to this R3V6-GA solution under constant stirring, followed by a second sonication at 70 W for 20 min to complete the formation of micelles. The final concentration of the AMO155c-R3V6-GA micelles was adjusted to 0.84 mg/ml.

### Transmission electron microscopy

Transmission electron microscopy (TEM) was used to investigate the morphology and size of the nanovesicles. Mesh copper grids (Ted Pell, Redding, CA) were rinsed with droplets of distilled water. Then, AMO155c-R3V6-GA was then loaded onto the grids and incubated for 30 min at 37 °C until the samples dried. For negative staining, 2% uranyl acetate was applied. The samples were observed using TEM (NEOARM, JEOL, Tokyo, Japan).

### Intracellular uptake of AMO155c: Flow cytometry

L2 lung epithelial cells were cultured in RPMI 1640 media containing 10% fetal bovine serum. L2 cells were seeded in a 12-well plate at 1 × 10^5^ cells per well. To optimize the ratio between AMO155c and R3V6, Cy5-labeled AMO155c (Cy5-AMO155c) was mixed with various amounts of R3V6. Cy5-AMO155c-R3V6 binary complex was added to the cells at a final concentration of 2 μg of AMO155c per well. After 24 h of incubation, Cy5-positive cells were evaluated by flow cytometry to optimize the AMO155c-R3V6 ratio.

To optimize the ratio of AMO155c and GA, AMO155c-R3V6-GA micelles were prepared with increasing ratios of GA. The AMO155c-R3V6-GA micelles were transfected into L2 cells. The intracellular delivery of the AMO155c-R3V6-GA micelles was measured by flow cytometry.

To evaluate the cholesterol moiety of AMO155c, AMO155 without a cholesterol moiety was used as a control. AMO155c-R3V6-GA micelles and AMO155-R3V6-GA complexes were prepared with Cy5-AMO155c or Cy5-AMO155, respectively. AMO155-R3V6-GA and AMO155c-R3V6-GA were prepared by mixing 2 μg of AMO155/AMO155c, 20 μg of R3V6, and 20 μg of GA in 50 μl of 5% glucose solution at a concentration of 0.84 mg/ml. The AMO155c-R3V6-GA micelles were added to L2 cells and intracellular delivery was measured by flow cytometry.

### Fluorescence microscopy study

L2 cells were seeded on a chamber slide at a density of 5 × 10^4^ cells per well. AMO155c-R3V6-GA were transfected into the cells as described above. The cells were washed twice with Dulbecco’s phosphate-buffered saline (DPBS), and the nuclei were stained with DAPI. The slides were mounted and observed using Cellena S (Logos Biosystems, Gyeonggi-do, South Korea) digital imaging system.

### Endocytosis inhibitor tests to determine the intracellular uptake mechanism of AMO155c-R3V6-GA micelles

L2 cells were plated in a 12-well plate at a concentration of 1 × 10^5^ cells per well. Endocytosis inhibitor tests were performed as described previously [32,33].

### Critical micelle concentration

R3V6, GA, R3V6-GA, and AMO155c-R3V6-GA solutions were prepared at various concentrations. Critical micelle concentration (CMC) was determined as described previously [15].

### Micelle size and zeta potential

The sizes and zeta potentials of the AMO155c-R3V6 and AMO155c-R3V6-GA micelles were evaluated using a Zetasizer (Malvern Instruments, Worcestershire, UK).

### Gel retardation assays and heparin competition assay

Fixed amounts of AMO155c or AMO155 (0.5 μg) were mixed with increasing amounts of R3V6 or GA in a 5% glucose solution. Gel retardation assays were carried out as described previously [34]. The stabilities of the micelles AMO155c-R3V6, AMO155-R3V6, and AMO155c-R3V6-GA were analyzed by heparin competition assay as described previously [34].

### In vitro cytokine ELISA

Raw264.7 cells were seeded into 12-well plates at 1 × 10^5^ cells per well. The cells were cultured for 24 h. AMO155c-R3V6 and AMO155c-R3V6-GA micelles were prepared using optimal conditions. LPS (20 ng) was added to the cells. The cells were incubated with the AMO155c-R3V6 and AMO155c-R3V6-GA micelles at 37 °C for 4 h. After removal of transfection mixture, the cells were cultured in fresh Dulbecco’s modified Eagle medium for an additional 24 h. The culture media were subjected to ELISA according to the manufacturer’s manual.

### ALI mouse model

Animal experiments proceeded according to the guidelines of the Institutional Animal Care and Use Committee at Hanyang University (accreditation number: 2022-0135A). Male BALB/c mice (25 g) were used for animal experiments. After anesthetization, the mice were administered LPS (20 μg/mouse) in 5% glucose by intratracheal administration. Intratracheal administration is a method of administering drugs directly into the airway of a mouse. The anesthetized mouse was fixed in an upright position at a 90° angle to open the airway. Then, a syringe containing the drug was gently administered into the airway using a catheter. A strong light was shined on the neck of the mouse to make the airway visible (FOK-100W, Fiber Optic Korea, Daejeon, Korea). AMO155c-PEI25k, AMO155c-R3V6, and AMO155c-R3V6-GA were prepared at their optimal ratios in 50 μl of 5% glucose solution. The amount of AMO155c was fixed at 5 μg/mouse. The mice received the samples just after the instillation of LPS by intratracheal administration. After 24 h, the animals were sacrificed, and lung tissues and bronchoalveolar lavage (BAL) fluids were obtained for further analysis.

### In vivo cytokine ELISA

BAL fluid was centrifuged at 13,000 ×g and 4 °C to remove blood residues and cell debris. Then, the BAL fluid was subjected to ELISAs. The lung tissues were homogenized in reporter lysis buffer and centrifuged at 13,000 ×g. The supernatant was used in ELISAs.

### Hematoxylin and eosin staining

The tissue samples were fixed and stained with hematoxylin and eosin (H&E) as described previously [35]. Stained samples were observed using light microscopy after mounting.

### Immunohistochemistry

For immunohistochemistry, paraffin-embedded lung tissues were cut into 7-μm slices. The slices were stained with mouse monoclonal SOCS1 antibody. The nuclei were counterstained with DAPI and examined using a fluorescence microscope.

### Statistical analysis

Data were analyzed using analysis of variance, followed by the Newman–Keuls test. *P* values less than 0.05 were considered statistically significant.

## Results

### Preparation and optimization of AMO155c-R3V6-GA micelles for in vitro transfection

**Fig. 2.**
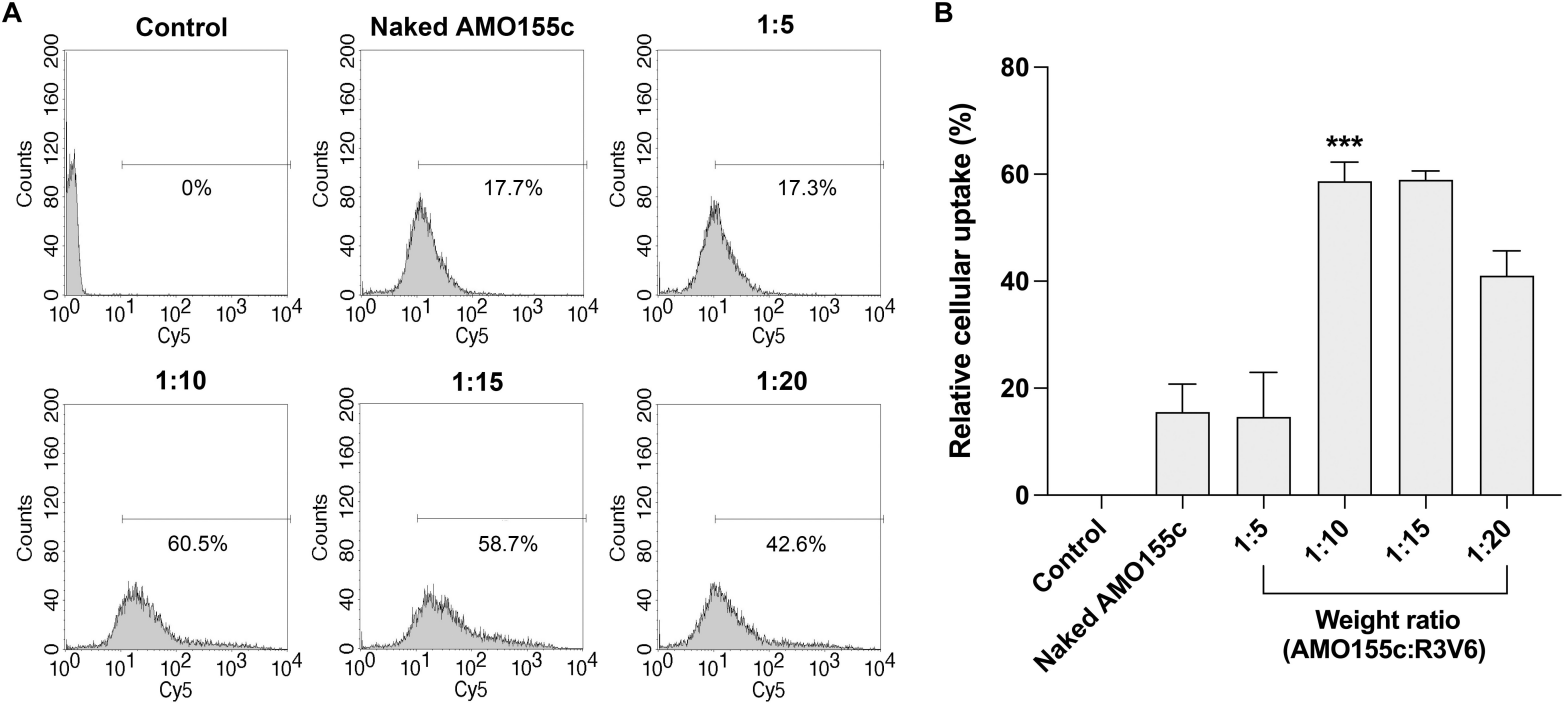
Intracellular delivery efficiency of AMO155c-R3V6 micelles, depending on the weight ratio. Cy5-AMO155c-R3V6 micelles were prepared at various weight ratios and transfected into L2 cells. Delivery efficiency was evaluated by flow cytometry. (A) Fluorescence intensity histograms of flow cytometry and (B) mean values of intracellular uptakes. The data are expressed as the mean ± standard deviation of quadruplicate experiments. ***P < 0.001 compared with control, naked AMO155c, 1:5, and 1:20.

**Fig. 3.**
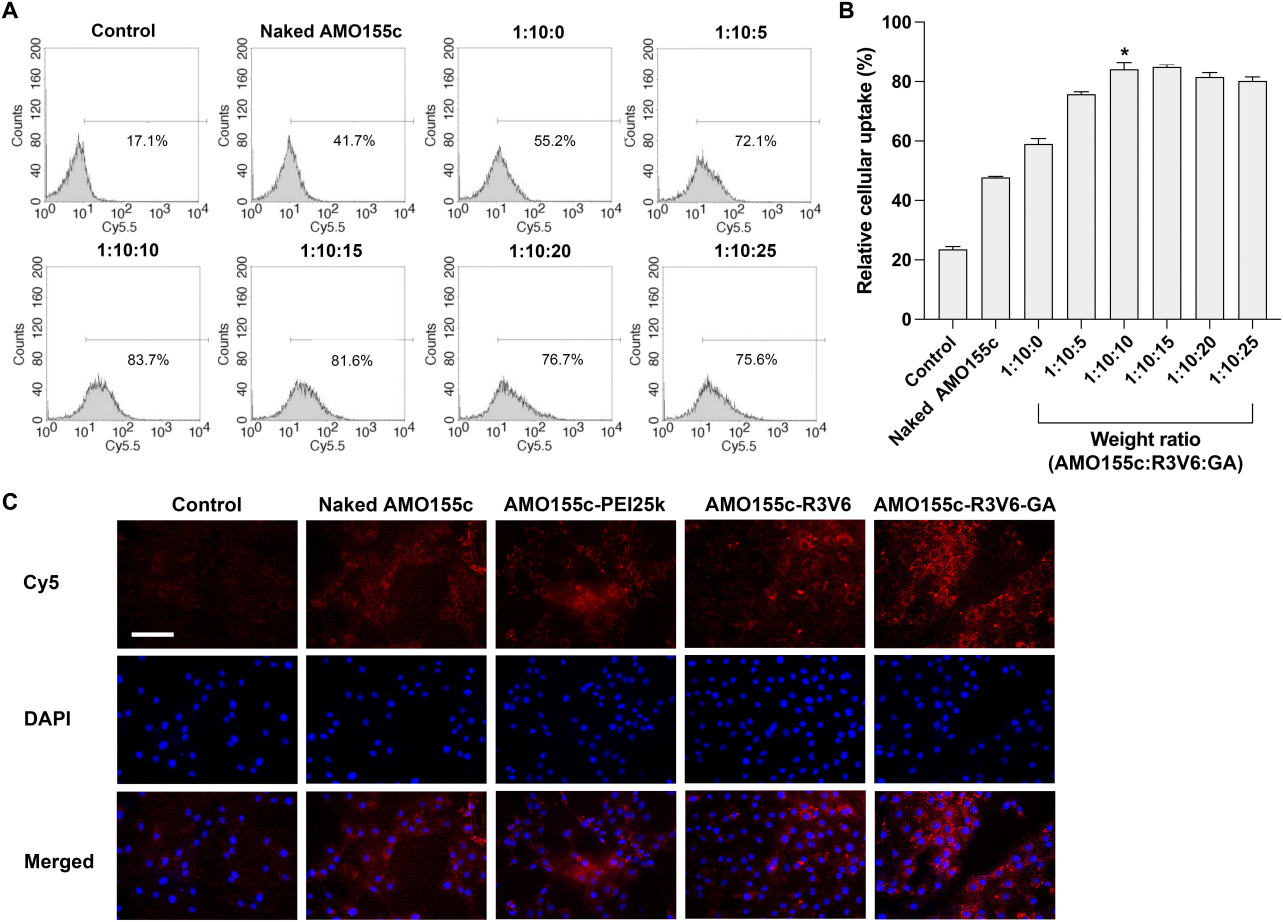
Intracellular delivery efficiency of AMO155c-R3V6-GA micelles. Cy5-AMO155c-R3V6-GA micelles were prepared at various weight ratios and transfected into L2 cells. Delivery efficiency was evaluated by flow cytometry. (A) Fluorescence intensity histograms of flow cytometry and (B) mean values of intracellular uptakes. The data are expressed as the mean ± standard deviation of quadruplicate experiments. *P < 0.05 as compared with control, naked AMO155c, 1:10:0, and 1:10:5. (C) Fluorescence microscopy. Cy5-AMO155c-carriers were prepared at their optimal ratios. The samples were added to the L2 cells. The cells were observed using a digital imaging system. Nuclei were counterstained with DAPI. The scale bar indicates 100 μm.

An aqueous solution of AMO155c-R3V6-GA ternary micelles was prepared with 3 components, AMO155c, R3V6, and GA (Fig. 1). To optimize the ratios of AMO155c, R3V6, and GA, delivery assays were conducted with L2 cells. First, the ratio between AMO155c and R3V6 was optimized by in vitro delivery assays. The AMO155c-R3V6 micelles at different weight ratios were delivered into L2 cells. The results showed that the micelles at ratios of 1:10 and 1:15 had higher delivery efficiency than the micelles at other ratios (Fig. 2A and B). There was no statistical significance between the 1:10 and 1:15 ratios. To minimize the possible cytotoxicity of R3V6, a weight ratio of 1:10 was used to prepare AMO155c-R3V6 micelles for following experiments. Then, various amounts of GA were mixed to prepare AMO155c-R3V6-GA micelles to optimize the ratio of GA to AMO155c. The in vitro delivery assays indicated that the highest delivery efficiency was obtained at weight ratios of 1:10:10 (AMO155c:R3V6:GA) (Fig. 3A and B). In correspondence to these results, the ratio of AMO155c-R3V6-GA micelles was fixed at 1:10:10.

**Fig. 4.**
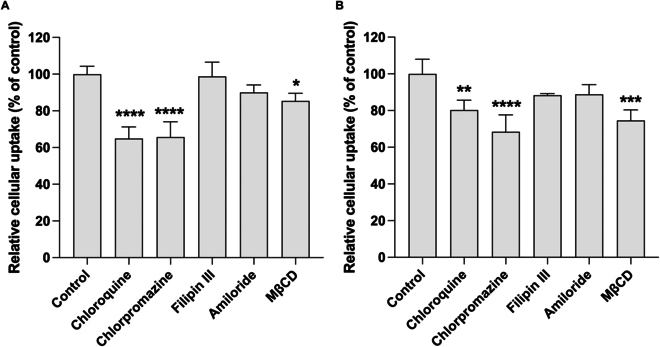
Mechanism of cellular uptake. (A) Cy5-AMO155c-R3V6 and (B) Cy5-AMO155c-R3V6-GA. micelles were prepared at their optimal weight ratios and transfected into L2 cells. The transfection was performed in the presence of chloroquine, chlorpromazine, filipin III, amiloride, and MβCD. The delivery efficiency was measured by flow cytometry. The data are presented as the mean ± standard deviation of quadruplicate experiments. *P < 0.05, **P < 0.01, and ****P < 0.0001 compared with control.

To evaluate the delivery efficiency, AMO155c-R3V6-GA micelles were delivered to L2 cells and observed by fluorescence microscopy (Fig. 3C). Naked AMO155c, AMO155c-PEI25k, and AMO155c-R3V6 complexes were used as controls. The fluorescence microscopy results indicated that AMO155c-R3V6-GA had higher delivery efficiency than the controls (Fig. 4).

The cytotoxicity of the AMO155c-R3V6 and AMO155c-R3V6-GA micelles was evaluated by using MTT assays (Fig. S1). The viability of L2 cells was only decreased by R3V6 alone, suggesting that R3V6 alone may induce cytotoxicity. The toxicity of the R3V6 peptide may be due to the positive charge of the peptides. The R3V6 peptides might interact with the negative cell surface, inducing hydrophobic collapse and membrane rupture [36]. However, a previous study found that the toxicity of R3V6 was much less than that of a representative cationic polymeric carrier, PEI25k [20,21]. The toxicities of the AMO155c-R3V6 and AMO155c-R3V6-GA micelles were similar to R3V6 alone, suggesting that the micelles might not induce severe toxicity, unlike PEI25k.

The endocytosis mechanism of the AMO155c-R3V6 and AMO155c-R3V6-GA micelles was verified by in vitro delivery assays in the presence of various endocytosis inhibitors. Chlorpromazine could reduce the delivery efficiency of AMO155c-R3V6 (Fig. 4A). Therefore, the intracellular delivery of AMO155c-R3V6 micelles may follow a clathrin-mediated endocytosis pathway. Chloroquine also reduced transfection efficiency (Fig. 4A). This may be because chloroquine is an inhibitor of autophagy, blocking autophagosome fusion with lysosomes. This can affect the function of clathrin and clathrin-coated vesicles, resulting in a decrease in clathrin-dependent endocytosis [37,38]. In addition, it can inhibit the progression from type 2 coated pits to type 3 [38,39]. The inhibition by chloroquine also indicated that cellular entry of the AMO155c-R3V6-GA micelles may be through clathrin-mediated endocytosis. Cholesterol depletion by MβCD also reduced delivery efficiency slightly, suggesting that cholesterol-raft-dependent endocytosis may contribute to the delivery of the micelles (Fig. 4A). In vitro delivery assays with AMO155c-R3V6-GA micelles showed similar results to AMO155c-R3V6 micelles (Fig. 4B). Therefore, clathrin-mediated endocytosis and cholesterol-raft-dependent endocytosis may contribute to the delivery efficiency of the AMO155c-R3V6-GA micelles.

### Physical characterization of AMO155c-R3V6-GA micelles

**Fig. 5.**
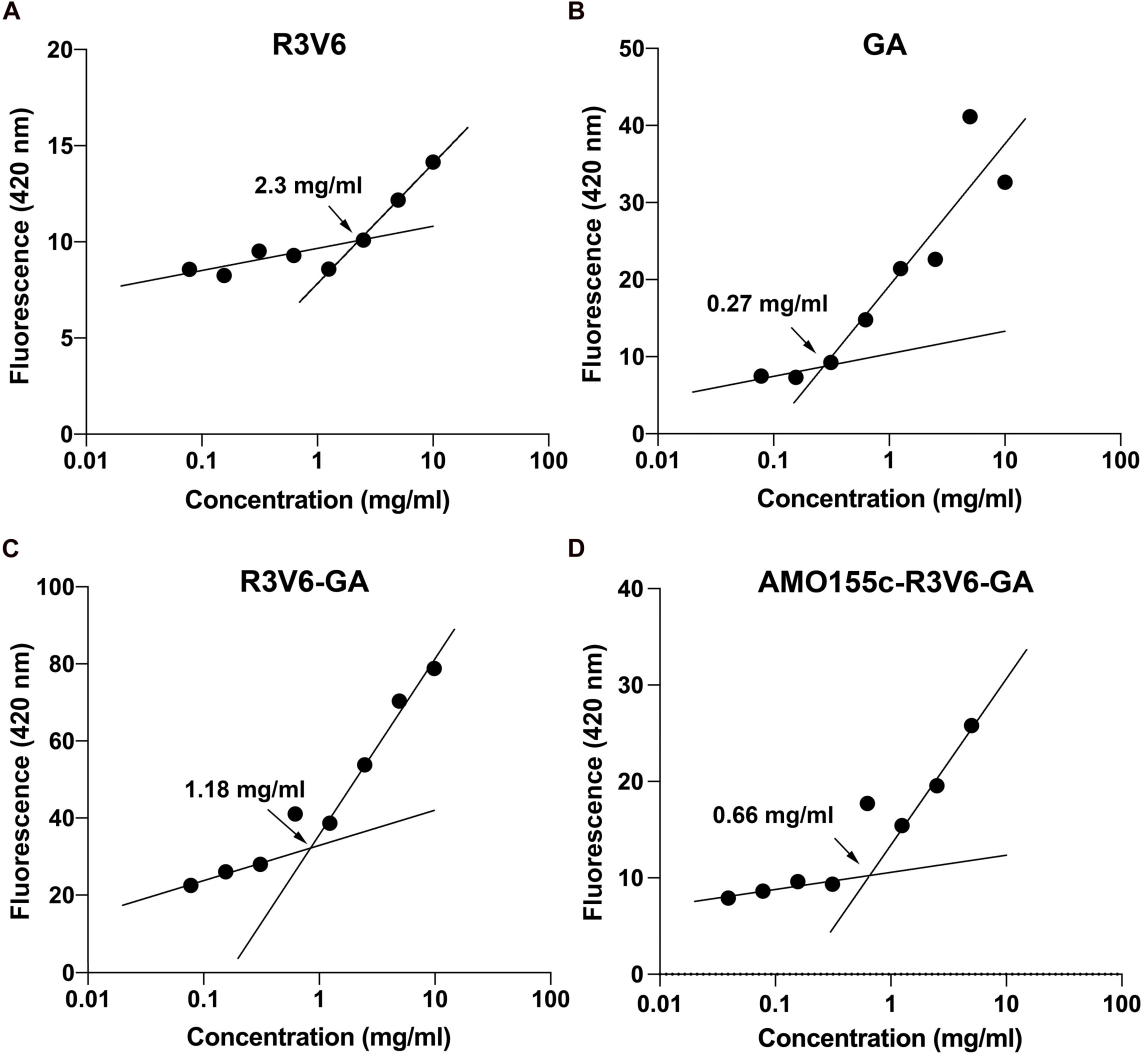
CMC. The CMCs of (A) R3V6, (B) GA, (C) R3V6-GA, and (D) AMO155c-R3V6-GA. were measured by the DPH method.

The amphiphilic properties of R3V6 and GA allow the formation of micellar structures in aqueous solution. To confirm micelle formation, CMCs of R3V6, GA, and R3V6/GA were measured by the DPH method. R3V6 and GA had CMCs of 2.3 and 0.27 mg/ml, respectively (Fig. 5A and B). The mixture of R3V6 and GA was prepared at a 1:1 weight ratio because AMO155c-R3V6-GA micelles were prepared at this ratio. The CMC of the R3V6/GA mixtures was 1.18 mg/ml, which was higher than GA and lower than R3V6 (Fig. 5C). The CMC of AMO155c-R3V6-GA was 0.66 mg/ml. In AMO155c-R3V6-GA micelles, AMO155c may stabilize the micelles by charge interactions as well as hydrophobic interactions (Fig. 5D).

**Fig. 6.**
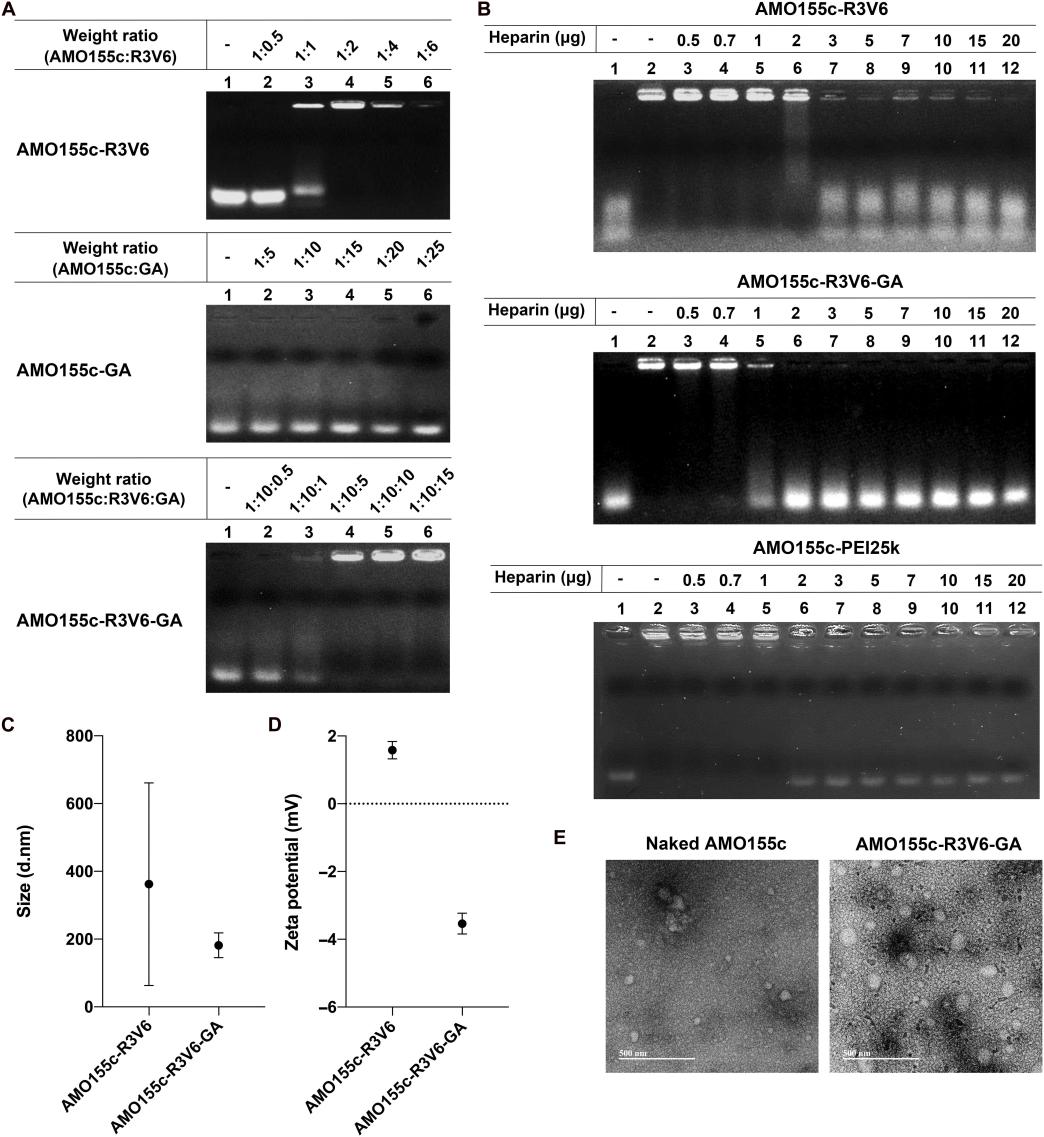
Physical characterization. (A) Gel retardation assay. AMO155c was mixed with various amounts of R3V6, GA, or R3V6-GA and analyzed on a 1% agarose gel. (B) Heparin competition assay. AMO155c-R3V6, AMO155c-R3V6-GA, and AMO155c-PEI25k were prepared at their optimal ratios for transfection. Increasing amounts of heparin were added to the mixtures. The samples were analyzed on a 1% agarose gel. (C) Particle size and (D) zeta potential. The sizes and zeta potentials of the micelles were measured by a Zetasizer. The data are expressed as the mean ± standard deviation of quadruplicate experiments. (E) TEM image. AMO155c-R3V6-GA micelles were prepared at a 1:10:10 weight ratio. The morphology was observed by TEM. The scale bar indicates 500 nm.

Micelle formation was also analyzed by gel retardation assays. As a result, the AMO155c was retarded at a weight ratio of 1:2 (Fig. 6A). The binding of AMO155c and R3V6 was through not only hydrophobic interactions but also charge interactions. Charge interactions between AMO155c and R3V6 may neutralize the negative charges of AMO155c, resulting in the reduced mobility of AMO155c in the gel. Also, AMO155c and GA mixtures were analyzed by agarose gel electrophoresis. In this case, AMO155c was not retarded at all by mixing it with GA (Fig. 6A). Both AMO155c and GA have negative charges and may induce repulsion between them, interfering with micelle formation. In the mixture of AMO155c, R3V6, and GA, AMO155c was retarded completely in the gel at 1:10:5 (Fig. 6A). In the AMO155c-R3V6-GA micelles, the positive charge of R3V6 may participate in binding to AMO155c and GA. Therefore, it seems that both charge and hydrophobic interactions may contribute to the stability of AMO155c-R3V6-GA micelles.

The stability of AMO155c-R3V6-GA micelles was measured in the presence of excess amounts of polyanionic polymers by heparin competition assays. AMO155c of AMO155c-R3V6 micelles was released from the micelles and migrated in the agarose gel in the presence of 2 mg of heparin (Fig. 6B). In contrast, AMO155c of AMO155c-R3V6-GA micelles was released with 1 mg of heparin (Fig. 6B). This indicated that the AMO155c-R3V6-GA micelles might be less stable than the AMO155c-R3V6 micelles. This may be due to the negative charge of GA, which reduced the charge interaction between AMO155c and R3V6. The stability of AMO155c-R3V6-GA micelles was similar to that of the AMO155c-PEI25k complex (Fig. 6B). Considering that PEI25k is a widely used polymeric carrier, it seems that AMO155c-R3V6-GA micelles may have enough stability for the delivery of therapeutic oligonucleotides.

The particle sizes of AMO155c-R3V6 and AMO155c-R3V6-GA micelles were around 390 and 200 nm, respectively (Fig. 6C). The surface charges were positive 1.8 mV and negative 3.6 mV, respectively (Fig. 6D). TEM images indicated that naked AMO155c and AMO155c-R3V6-GA formed spherical nanoparticles (Fig. 6E). In particular, the negative surface charge of AMO155c-R3V6-GA micelles may be beneficial for delivery into the lungs. Positively charged particles are easily captured and cleared in the lungs because of the negative mucin in the mucus layer. Thus, negatively charged AMO155c-R3V6-GA micelles may avoid this clearance by mucin and easily reach epithelial cells in the lungs.

### Comparison of AMO155 and AMO155c

**Fig. 7.**
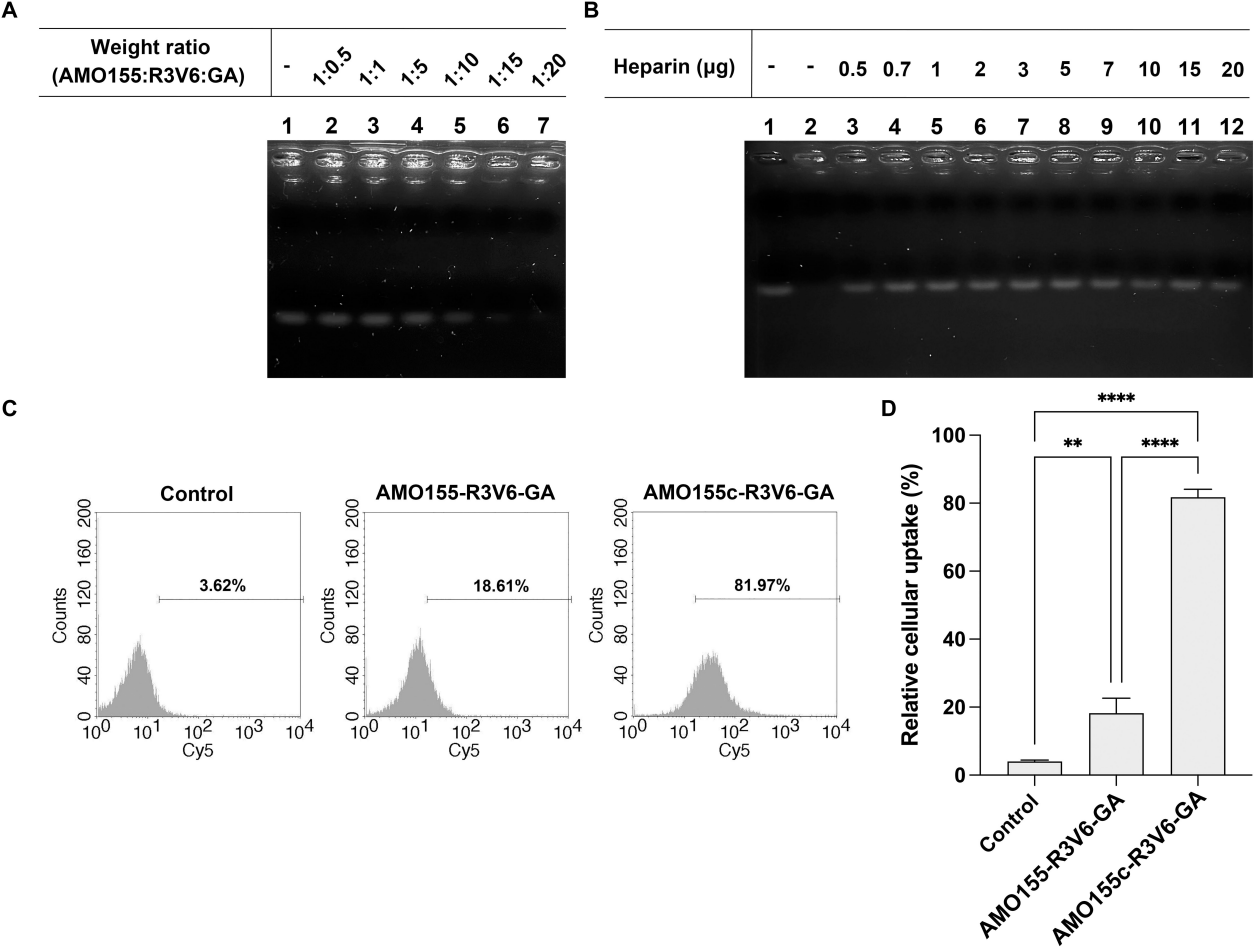
Comparison of AMO155c with AMO155. (A) Gel retardation assay with AMO155 and R3V6. AMO155 was mixed with various amounts of R3V6 and analyzed on a 1% agarose gel. (B) Heparin competition assay with AMO155-R3V6-GA. AMO155-R3V6-GA complex was mixed with increasing amounts of heparin and analyzed on a 1% agarose gel. (C) Fluorescence intensity histograms of flow cytometry and (D) Mean values of intracellular uptakes. Cy5-AMO155-R3V6-GA and Cy5-AMO155c-R3V6-GA were prepared and transfected into L2 cells. The cellular uptake of AMO155 or AMO155c was measured by flow cytometry. The data are expressed as the mean ± standard deviation of quadruplicate experiments. **P < 0.01, ****P < 0.0001.

The cholesterol moiety of AMO155c may contribute to the formation of AMO155c-R3V6-GA micelles. To evaluate the effect of the cholesterol moiety, we compared AMO155, which did not have a cholesterol moiety, with AMO155c. In a gel retardation assay, AMO155 was not retarded at a weight ratio of 1:15 (AMO155:R3V6) (Fig. 7A). In contrast, AMO155c was retarded completely at a 1:2 weight ratio (Fig. 6A). This suggests that the AMO155-R3V6 complex may be formed by charge interactions without hydrophobic interactions, and therefore, a larger amount of R3V6 is required to retard AMO155 in the gel.

The stability of the AMO155-R3V6-GA complex was compared with that of AMO155c-R3V6-GA micelles by heparin competition. In the AMO155-R3V6-GA complex, AMO155 began to be released in the presence of 0.5 μg of heparin (Fig. 7B). In contrast, AMO155c-R3V6-GA micelles did not release AMO155c in the presence of 0.5 μg of heparin (Fig. 6B). This result indicated that the cholesterol moiety of AMO155c contributes to the formation and stability of AMO155c-R3V6-GA micelles by hydrophobic interactions.

The efficiency of the delivery may be dependent on the stability of AMO155c-R3V6-GA micelles. To evaluate this effect, the AMO155-R3V6-GA complex was delivered into L2 cells, and the results were compared with that of AMO155c-R3V6-GA micelles. The intracellular delivery efficiency of AMO155-R3V6-GA was lower than that of AMO155c-R3V6-GA micelles (Fig. 7C and D). Therefore, the cholesterol moiety of AMO155c contributes to the format ion of AMO155c-R3V6-GA micelles and a higher delivery efficiency.

### In vitro anti-inflammatory effects of AMO155c-R3V6-GA micelles

The delivery of the AMO155c-R3V6-GA micelles may induce an anti-inflammatory effect due to the combined effects of AMO155c and GA. AMO155c may inhibit the activity of miRNA155, resulting in a reduction of proinflammatory cytokines. GA is a natural anti-inflammatory drug, which inhibits NF-κB and decreases the production of proinflammatory cytokine.

**Fig. 8.**
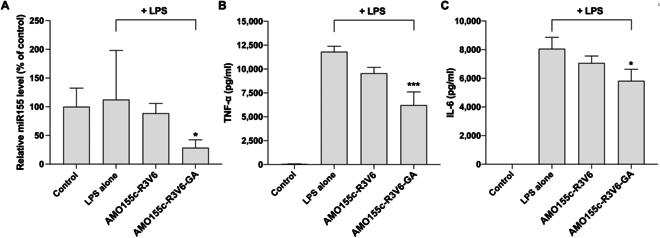
Effects of AMO155c-R3V6-GA on inflammatory cytokines in vitro. AMO155c-R3V6 and AMO155c-R3V6-GA micelles were prepared at their optimal weight ratios and transfected into LPS-activated Raw264.7 cells. (A) The miRNA155 levels in the cells were evaluated by reverse transcription polymerase chain reaction. The data are expressed as the mean ± standard deviation of quadruplicate experiments. *P < 0.05 compared with control. The levels of TNF-α (B) and IL-6 (C) in the culture media were measured by ELISAs. The data are expressed as the mean ± standard deviation of quadruplicate experiments. *P < 0.05 compared with control and LPS alone. ***P < 0.001 compared with control, LPS alone, and AMO155c-R3V6.

Real-time reverse transcription polymerase chain reaction was performed to measure the miRNA155 level after the delivery of AMO155c. The results indicated that the miRNA155 level was decreased by the AMO155c-R3V6-GA micelles more effectively than AMO155c-R3V6 micelles (Fig. 8A). In Fig. 3, AMO155c-R3V6-GA micelles had a higher AMO155c delivery efficiency than AMO155c-R3V6 micelles. This explains why AMO155c-R3V6-GA micelles had a higher inhibitory effect than AMO155c-R3V6 micelles.

The AMO155c-R3V6 and AMO155c-R3V6-GA micelles were added to LPS-activated Raw264.7 cells and cytokine ELISAs were performed with cell culture media. The results indicated that AMO155c-R3V6 micelles reduced TNF-α and IL-6 levels (Fig. 8B and C). A possible explanation is that AMO155c delivered by AMO155c-R3V6 micelles reduces miRNA155 and decreases the production of proinflammatory cytokines. However, the cytokine levels were further decreased by the treatment of AMO155c-R3V6-GA micelles (Fig. 8A and B). The higher anti-inflammatory effects induced by AMO155c-R3V6-GA micelles compared to AMO155c-R3V6 micelles may be explained by 2 factors. First, AMO155c-R3V6-GA micelles deliver AMO155c more efficiently than AMO155c-R3V6 micelles. Therefore, the inhibition effect of miRNA155 was greater in the cells treated with AMO155c-R3V6-GA micelles than with AMO155c-R3V6 micelles. Second, GA in the AMO155c-R3V6-GA micelles contributes to additional anti-inflammatory effects.

### Therapeutic effects of AMO155c-R3V6-GA micelle delivery in ALI animal models

**Fig. 9.**
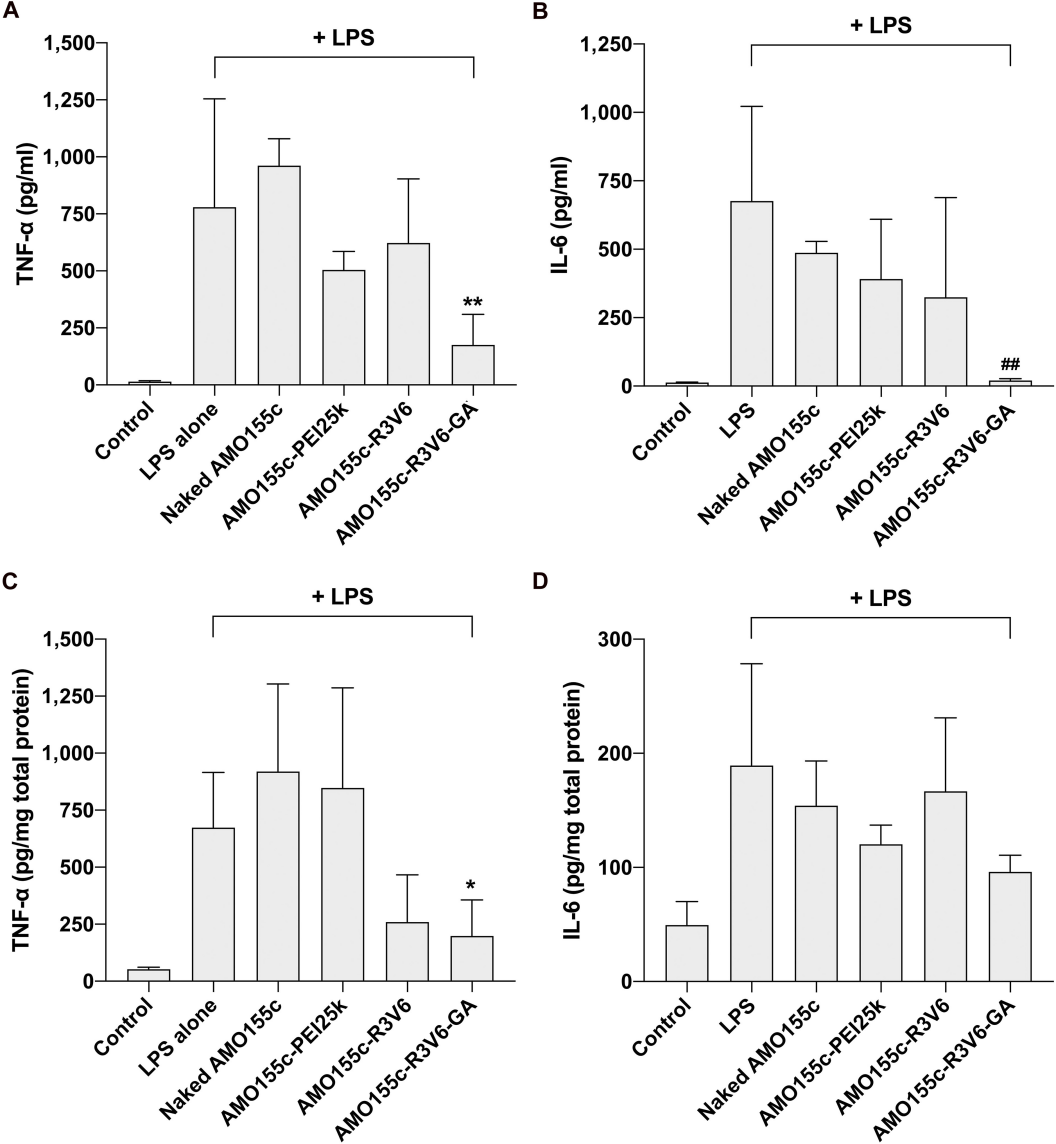
Effects of AMO155c-R3V6-GA on inflammatory cytokines in ALI models in vivo*.* AMO155c-PEI25k, AMO155c-R3V6, and AMO155c-R3V6-GA micelles were prepared at their optimal weight ratios and administered into the ALI models by intratracheal instillation. After 24 h, the lungs and BAL fluids were obtained for further analysis. The BAL fluids were subjected to (A) TNF-α and (B) IL-6 ELISAs. The data are expressed as the mean ± standard deviation of quadruplicate experiments. **P < 0.01 compared with LPS alone, naked AMO155c, and AMO155c-PEI25k. ^##^*P* < 0.01 compared with LPS and naked AMO155c. Tissue extracts were subjected to (C) TNF-α and (D) IL-6 ELISAs. The data are expressed as the mean ± standard deviation of quadruplicate experiments. *P < 0.05 compared with LPS alone, naked AMO155c, and AMO155c-PEI25k.

The AMO155c-R3V6-GA micelles were prepared and administered into the lungs of ALI mouse models. After 24 h, the BAL fluid and lung tissues were obtained and subjected to ELISAs to evaluate the cytokine levels. LPS treatment increased the TNF-α and IL-6 levels in the BAL fluid, suggesting that inflammatory reactions increased substantially (Fig. 9A and B). The administration of naked AMO155c, AMO155c-PEI25k, and AMO155c-R3V6 micelles tended to decrease the cytokine levels, although the differences were not statistically significant compared with LPS alone. However, AMO155c-R3V6-GA micelles decreased the TNF-α and IL-6 levels the most effectively (Fig. 9A and B). This effect was confirmed by the ELISA of lung tissue extracts. In the lung tissues, AMO155c-R3V6-GA micelles reduced the TNF-α level effectively (Fig. 9C). The IL-6 ELISA of tissue extracts tended to indicate reduced levels of IL-6 in the AMO155c-R3V6-GA samples, although the difference was not statistically significant (Fig. 9D). Therefore, the results suggest that AMO155c-R3V6-GA micelles may decrease the inflammatory response in ALI animal models by a reduction of proinflammatory cytokines.

To evaluate the anti-inflammatory effect of GA alone, GA alone was administered into the ALI models and compared with the results of AMO155c-R3V6-GA (Fig. S2). The results indicated that GA alone reduced the proinflammatory cytokine in the ALI models, compared with the LPS control. However, AMO155c-R3V6-GA decreased the cytokine level further, suggesting that the micelles had higher anti-inflammatory effect than GA alone.

**Fig. 10.**
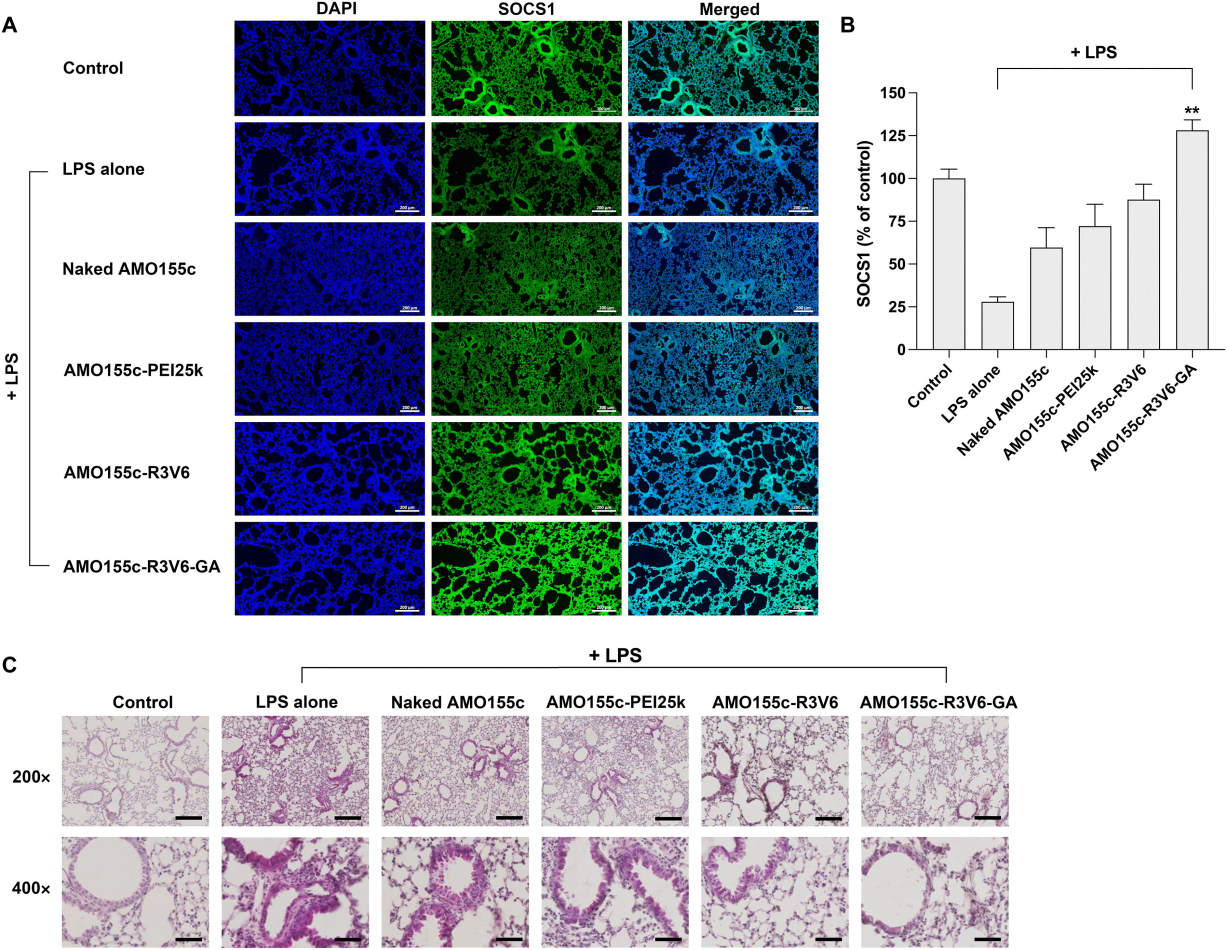
Immunohistochemistry and H&E staining. AMO155c-PEI25k, AMO155c-R3V6, and AMO155c-R3V6-GA micelles were prepared at their optimal weight ratios and administered into the ALI models by intratracheal instillation. After 24 h, the lungs were obtained from the animals. (A) Immunohistochemistry. The lung tissues were subjected to SOCS1 immunohistochemistry. The scale bar represents 200 μm. (B) Quantitation of SOCS1 levels. The quantitation of the SOCS1 level was performed with Zeiss Zen software. The data are expressed as mean value ± standard deviation of quadruplicate experiments. **P < 0.01 compared with the other groups. (C) H&E staining. The lungs were obtained and subjected to H&E staining. The samples were observed by optical microscopy. The scale bars indicate 200 μm in 200× and 50 μm in 400×.

The anti-inflammatory effect of AMO155c is closely related to SOCS1, which is an inhibitor of the cytokine signaling pathway [25]. Overexpression of miRNA155 in ALI facilitates the degradation of SOCS1 mRNAs, reducing its expression. Since SOCS1 is an inhibiting protein of NF-κB, a key transcription factor in the expression of proinflammatory cytokines, the decrease of SOCS1 results in the induction of proinflammatory cytokines such as TNF-α and IL-6. Thus, the reduction of proinflammatory cytokines may be due to an increase of SOCS1 by AMO155c. The expression level of SOCS1 was evaluated by immunostaining (Fig. 10A and B). The results indicated that the expression of SOCS1 was increased by AMO155c-R3V6-GA micelles the most effectively (Fig. 10A and B).

The anti-inflammatory effects of AMO155c-R3V6-GA micelles were confirmed by the H&E staining of the lung tissues. Compared with LPS alone, the AMO155c-R3V6-GA samples reduced the width of the septum, hemolysis, and infiltration of monocytes into the lungs (Fig. 10C). The results suggest that the damage and immune response in the lungs of ALI models are effectively reduced by the AMO155c-R3V6-GA micelles.

## Discussion

In this study, we have shown that cholesterol-conjugated AMO155 formed stable ternary micelles with R3V6 and GA and facilitated the pulmonary delivery of AMO155 and GA into the inflammatory lungs by intratracheal administration. In vitro and in vivo delivery assays showed that AMO155c-R3V6-GA ternary micelles delivered AMO155 and GA into the lungs effectively. In addition, AMO155 and GA had synergistic effects in delivery and therapeutic effects in the ALI animal models.

In the previous study, R3V6 was characterized as a delivery carrier of antisense oligonucleotides [40]. In this report, R3V6s formed complexes with AMOs by charge interaction. However, the stability AMO-R3V6 was not strong enough for pulmonary or systemic delivery, releasing AMOs easily in the presence of negatively charged molecules. In particular, mucins in the mucus layer may facilitate the release of AMOs from the complexes. To increase the stability of AMO/R3V6, cholesterol-conjugated AMOs were used to prepare AMOc-R3V6 comicelles. In vitro characterization showed that AMO155c formed stable micelles with R3V6 than AMO155. The results indicated that cholesterol moiety of AMO155c played a key role in formation of micelles. In gel retardation assays, AMO155-R3V6-GA micelles were completely retarded with 15× weight excess R3V6, while AMO155c-R3V6-GA micelles with 5× weight excess R3V6 (Figs. 6A and 7A). Thus, this indicated clearly that AMO155c-R3V6-GA formed stable micelles with less amount of R3V6 than AMO155. Due to the cholesterol moiety of AMO155c, AMO155c-R3V6-GA was more stable in the presence of heparin than AMO155-R3V6-GA (Figs. 6B and 7B). This suggests that the core of the micelles with cholesterols may be more hydrophobic, contributing to more efficient integration of GA into AMO155c-R3V6. As such, intracellular delivery efficiency of AMO155c-R3V6-GA was higher than that of AMO155-R3V6-GA (Fig. 7C and D).

In vivo experiments indicated that the proinflammatory cytokines were decreased by AMO155c-R3V6-GA (Fig. 9). The reduction of cytokines can be explained by 2 factors. First, the anti-inflammatory effects of AMO155c-R3V6-GA may be caused by the anti-inflammatory effect of GA. GA is a natural anti-inflammatory drug, which has been widely used for various diseases [41]. GA decreases NF-κB activity, resulting in a reduction of proinflammatory cytokine production. Second, the increased expression of SOCS1 by AMO155c inhibits the activity of NF-κB. Therefore, GA may have a synergistic effect with AMO155c in anti-inflammatory therapy.

In addition to anti-inflammatory effects, GA has additional function in delivery of AMO155c into the lungs. First, GA may improve the delivery efficiency of AMO155c-R3V6 micelles by masking a positive charge, which facilitates the penetration of the mucus layers. Second, the cellular receptors of GA, which have not been identified, may increase the uptake of micelles containing GA by receptor-mediated endocytosis. In a previous study, pDNA-polymer complexes had higher transfection efficiency in the presence of GA, compared with the complexes without GA in vitro and in vivo [31]. This may be due to the receptor-mediated endocytosis of the complexes containing GA [31]. Therefore, GA is not only a natural anti-inflammatory drug but also an enhancer or facilitator of intracellular delivery of ternary micelles in the lung cells.

AMO155 has been evaluated as a therapeutic option for ALI [27,42]. Delivery of AMO155 into lungs was achieved with PAMAM-entrapped gold nanoparticles in previous study [42]. The study indicated that PAMAM delivered AMO155 into the LPS-activated alveolar macrophages, down-regulating proinflammatory cytokines. However, the combination with dexamethasone had higher therapeutic effects in the ALI models. In this study, dexamethasone was covalently linked to PAMAM by chemical reactions. In our current study, GA was used as an anti-inflammatory drug for combination therapy. GA was incorporated into the AMO155c-R3V6 micelles for enhanced therapeutic effects. Unlike dexamethasone, GA has some advantages. First, GA can be easily incorporated into the AMO155c-based micelles in the simple mixture. In addition, GA has negative charge and can mask positive charges of AMO155c/R3V6 comicelles, which may facilitate penetration through the mucus layer. Furthermore, GA receptor-mediated endocytosis may increase the intracellular delivery efficiency of the micelles. Since AMO155c-R3V6-GA has a micelle structure, hydrophobic anti-inflammatory drug such as dexamethasone may be incorporated into the hydrophobic cores of the micelles as a future study.

In Fig. 7, AMO155c-R3V6-GA was prepared by mixing 2 μg of AMO155c, 20 μg of R3V6, and 20 μg of GA in 50 μl of 5% glucose solution. Therefore, the micelles were prepared at a concentration of 0.84 mg/ml, which was above CMC. Then, the micelle solution was added to the cells for intracellular delivery and diluted by cell culture medium. As a result, the final concentration of AMO155c-R3V6-GA was 0.08 mg/ml, which was below CMC. This suggested that the micelles might be dissociated in the solution. However, AMO155c-R3V6-GA was formed by charge interaction as well as hydrophobic interaction and might be more stable than the simple micelles formed by hydrophobic interaction. Thus, we speculate that the AMO155c-R3V6-GA micelles may be stable in the diluted solution, once they are formed in the concentrated solution. Due to the stability, AMO155c-R3V6-GA might have higher delivery efficiency than AMO155-R3V6-GA at this concentration (Fig. 7C and D).

In summary, ternary micelles composed of AMO155c, R3V6, and GA were investigated as therapeutic micelles for the efficient delivery of AMO155c and GA into an ALI animal model by intratracheal instillation. The results indicated that AMO155c-R3V6-GA ternary micelles increased the delivery efficiency of AMO155 oligonucleotides to the lungs and increased the anti-inflammatory effect. GA in the ternary micelles may have dual effects as an anti-inflammatory drug and an enhancer of AMO155c delivery. Based on these results, AMO155c-R3V6-GA micelles may be an efficient delivery platform for the delivery of AMO155c and GA for ALI therapy.

## Data Availability

The raw data supporting the conclusion of this article are made available upon request to the authors, without undue reservation.
