# Supplementary material for: Pulmonary Delivery of Anti-microRNA Oligonucleotide and Glycyrrhizic Acid Using Ternary Peptide Micelles for the Treatment of Acute Lung Injury
Source: Biomater Res. 2024 Nov 8;28:0107. doi: 10.34133/bmr.0107 (PMC11544319; doi:10.34133/bmr.0107)
Supplement: Supplementary 1 — Figs. S1 and S2 [file bmr.0107.f1.docx]

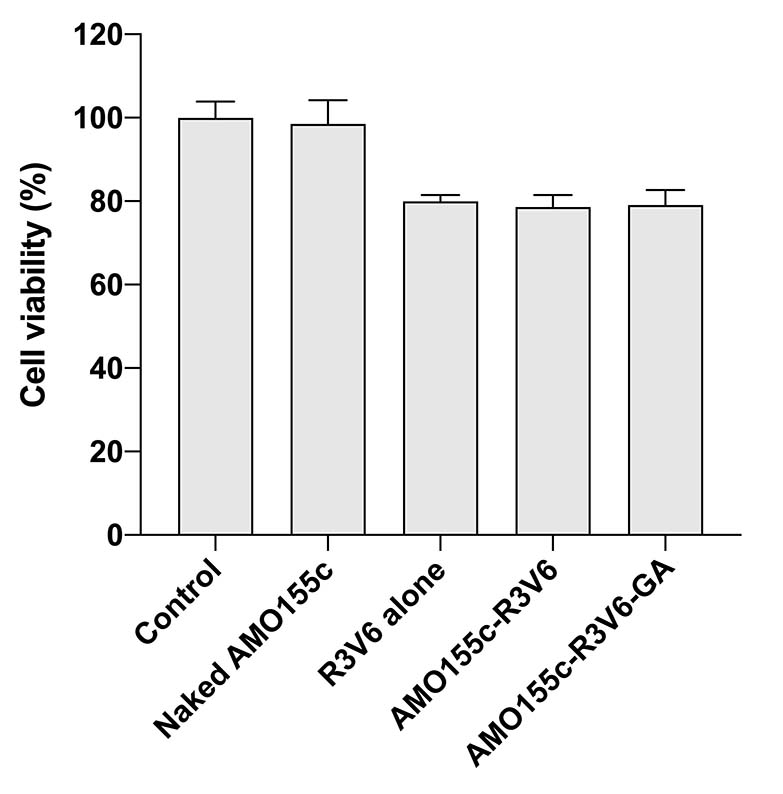


**Supplementary Figure 1. MTT assay.**

L2 cells were seeded in a 24-well plate at a density of 5×10^4^ cells/well. The cells were cultured in DMEM containing 10% FBS at 37°C for 24 h. To evaluate the toxicities of the micelles, naked AMO155c, R3V6 alone, AMO155c-R3V6, and AMO155c-R3V6-GA micelles were prepared and added to the cells. The amount of AMO155c was fixed at 0.5 μg/well. The cells were incubated with the samples for 4 h and the culture media were replaced with fresh DMEM containing 10% FBS. After incubation at 37°C for 24 h, 10 μg MTT/well was added and the cells were incubated at 37°C for 4 h. After the incubation, the media were removed and 700 μl DMSO was added to each well. The samples were incubated at 60°C for 5 min. The absorbance of the samples was measured at 570 nm using a microplate reader. Cell viability (%) = (OD_570_[sample]) / (average of OD_570_[control]) × 100.


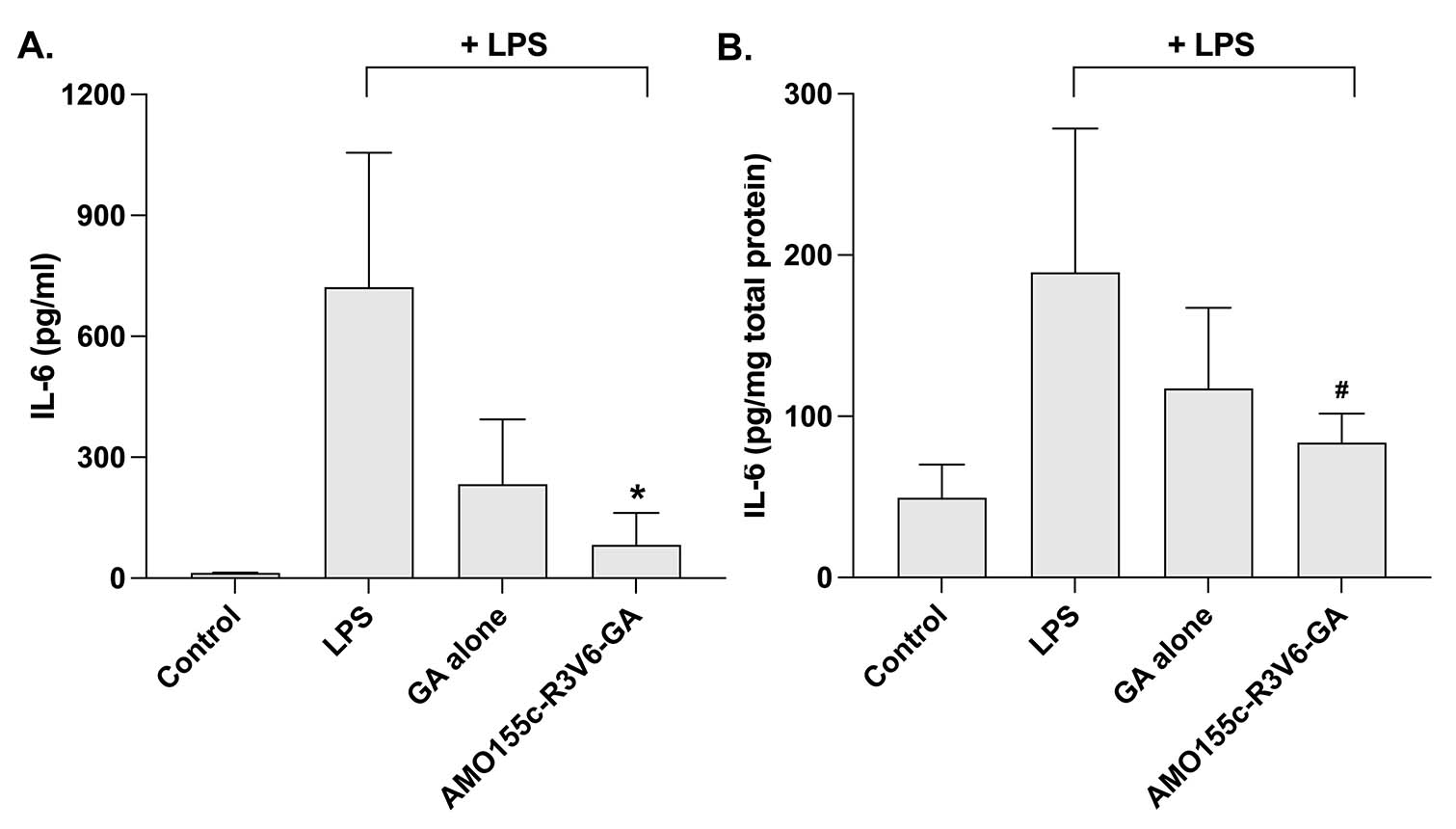


**Supplementary Figure 2. Comparison of AMO155c-R3V6-GA with GA alone in anti-inflammatory effects in ALI models *in vivo.***

GA alone and AMO155c-R3V6-GA micelles were prepared and administered into the ALI models by intratracheal instillation. After 24 h, the lungs and BAL fluids were obtained for further analysis. **(A) The BAL fluids and (B) tissue extracts** were subjected to IL-6 ELISAs. The data are expressed as the mean ± standard deviation of quadruplicate experiments. *P<0.05 compared with LPS and GA alone. ^#^P<0.05 compared with LPS.
